# Perceptions of Medications and Supplements by U.S. Dog‐Interested Members of the Public and Final‐Year Veterinary Students

**DOI:** 10.1111/jvp.70064

**Published:** 2026-03-09

**Authors:** Sidonie T. Gallinger, Johanna C. Heseltine, Kate Illing, Virginia R. Fajt, Dog Aging Project Consortium, Joshua M. Akey, Brooke Benton, Elhanan Borenstein, Marta G. Castelhano, Amanda E. Coleman, Kate E. Creevy, Kyle Crowder, Matthew D. Dunbar, Virginia R. Fajt, Annette L. Fitzpatrick, Unity Jeffery, Erica C. Jonlin, Matt Kaeberlein, Elinor K. Karlsson, Kathleen F. Kerr, Jonathan M. Levine, Jing Ma, Robyn L. McClelland, Daniel E. L. Promislow, Audrey Ruple, Stephen M. Schwartz, Sandi Shrager, Noah Snyder‐Mackler, M. Katherine Tolbert, Silvan R. Urfer, Benjamin S. Wilfond, Kate E. Creevy

**Affiliations:** ^1^ Veterinary Small Animal Hospital Texas A&M University College Station Texas USA; ^2^ Department of Small Animal Clinical Sciences Texas A&M College of Veterinary Medicine and Biomedical Sciences College Station Texas USA; ^3^ Department of Veterinary Physiology and Pharmacology Texas A&M College of Veterinary Medicine and Biomedical Sciences College Station Texas USA

**Keywords:** drug, education, nutraceutical, over‐the‐counter, prescription

## Abstract

Veterinary graduates must be prepared to educate clients about medications and supplements. We surveyed 1955 Dog Aging Project newsletter recipients and 40 final‐year veterinary students at Texas A&M College of Veterinary Medicine and Biomedical Sciences on their perceptions about medications and supplements. Respondents indicated whether each of 13 attributes applied to medications, supplements, neither, both, or “I'm not sure.” Frequency of responses by newsletter recipient respondents versus student respondents, respectively, were evaluated. We observed differences in the majority response for: (1) target a specific ailment (51% for newsletter recipients versus 62% for students, respectively); (2) target a specific condition (54% vs. 40%); (3) promote health and wellness (51% vs. 38%); (4) prevent worsening of a condition (60% vs. 72%); (5) are added to food (58% vs. 80%); (6) are recommended by a veterinarian (58% vs. 82%); (7) are covered by pet insurance (57% vs. 80%); and (8) are given to the animal long term or lifelong (55% vs. 72%). The overall distribution of responses was statistically significantly different between groups for three attributes: added to food (*p* < 0.001); recommended by a veterinarian (*p* = 0.005); and covered by pet insurance (*p* < 0.001). While a majority of both groups recognized that only medications are tested and approved by the Food and Drug Administration (FDA), 15% of final‐year veterinary students indicated that they thought both supplements and medications are FDA regulated, which suggests an important educational gap.

## 1 Introduction

Medications and supplements are mainstays of health in both human and veterinary medicine (Cowan et al. 2018; Djaoudene et al. 2023; Elrod and Hofmeister 2019; Finno 2020; Hoffman et al. 2022; Kramer et al. 2023; Tichy et al. 2024; Vandeweerd et al. 2012). With modern medicine's ever‐increasing ability to treat, mitigate, and prevent disease and to promote overall health and wellness, the ongoing use of both medications and supplements is expected. Selection of medications and supplements depends on a variety of factors, such as medical indications, patient comorbidities, and the potential for interactions and adverse effects (Bianco et al. 2020; Cowan et al. 2018; Goodman and Trepanier 2005; Koh et al. 2020; Satia‐Abouta et al. 2003). Another factor that impacts their application is users' perceptions and understanding of similarities and differences between medications and supplements.

Definitions for medications and supplements can be difficult to understand, particularly when applied to veterinary medicine. The Food and Drug Administration (FDA) defines a drug (medication) as a substance besides a food recognized by the United States Pharmacopeia, National Formulary, or Homeopathic Pharmacopeia of the United States intended to diagnose, treat, cure, prevent, or mitigate disease in man or animals (FDA 2025). The manufacture, sale, and marketing of medications are strictly regulated by the FDA and require extensive safety and efficacy data for approval. Medications can be subdivided into over‐the‐counter (OTC), prescription, or veterinary feed directive (FDA 2025). Medications that are FDA‐approved for human use can also be used for veterinary patients under regulations promulgated under extralabel drug use laws, such as the Animal Medicinal Drug Use Clarification Act (AMDUCA) (FDA—CFR 2024). The FDA's Dietary Supplement Health and Education Act (DSHEA) of 1994 defines a human dietary supplement as an ingested product that contains ingredient(s), such as vitamins, minerals, or herbs, that are intended to supplement the diet (FDA—Office of Dietary Supplement Programs 2024). Human supplements are marketed without FDA approval, and generally the FDA does not approve dietary supplement claims or other labeling before use. Manufacturers are responsible for ensuring their dietary supplements meet safety standards, and the FDA is limited to post‐market enforcement based on reported adverse events (FDA—Office of Dietary Supplement Programs 2024). There is no single regulatory definition of a veterinary supplement; the definition of human supplements cannot be carried over to veterinary supplements because the FDA does not consider the DSHEA to apply to companion animal supplements. While these supplements undergo less regulatory oversight, the general public may assume they are safe.

The National Academy of Sciences‐National Research Council (NAS/NRC) defines a pet supplement as a “substance for oral consumption by horses, dogs, and cats, whether in or on feed or offered separately, intended for the specific benefit to the animal by means other than provision of nutrients recognized as essential or for provision of essential nutrients for intended effect on the animal beyond normal nutritional needs, but not including legally defined drugs” (The National Academy of Sciences 2008). The FDA uses published information from the NAS/NRC to determine a standard level of supplementation (Finno 2020). In 2014, the NRC produced a report for the FDA on the safety (but not efficacy) of supplements, particularly lutein, evening primrose oil, and garlic for cats, dogs, and horses, which concluded that there was a lack of safety data (The National Academy of Sciences 2008).

The National Animal Supplement Council (NASC) advocates for Good Manufacturing Practice Quality Standards in veterinary supplements and awards a quality seal to products meeting their requirements (NASC 2025). NASC neither requires product efficacy studies nor verifies that scientific research data proving product efficacy are available. Additionally, there is no incentive for companies to have their products tested. Consumer Laboratory is a for‐profit laboratory through which manufacturers can voluntarily request testing of their products for a fee, with results only posted if the manufacturer consents. Consumer Laboratory validates ingredients but does not evaluate the efficacy of the supplement (ConsumerLab 2025; Finno 2020).

The use of alternate terminology such as “nutraceutical” to refer to some of these substances further complicates understanding. The American Veterinary Medical Association (AVMA) provides guidelines on the use of nutraceuticals, advising that veterinarian should be aware that animal nutritional supplements are typically not subject to premarketing evaluation by the FDA and should critically evaluate literature and other sources of information. Veterinarians should be aware of the ingredients; benefits, efficacy, and safety; and their legal liability if a recommended pet supplement or nutraceutical leads to adverse effects (AVMA 2001, 2003).

There is limited literature in human medicine regarding perceptions of medications and supplements. Positive perceptions toward medications generally include their necessity and benefits for health (Clyne et al. 2017; Horne and Weinman 1999; Phatak and Thomas 2006; Shiyanbola et al. 2013). Negative perceptions include concern for adverse effects, cost, and potential for polypharmacy (Clyne et al. 2017; Horne and Weinman 1999; Phatak and Thomas 2006; Shiyanbola et al. 2013). Positive perceptions toward supplements include the ability to slow or prevent disease, ability to enhance health, and perceived safety of the substance, while negative perceptions include concerns about efficacy and their marketing and regulation (Albright et al. 2012). The potential benefits and limitations may be perceived differently by individuals due to factors such as topic‐related education, cultural and societal influences, and personal experience (Albright et al. 2012).

Literature regarding perceptions about medications and supplements in veterinary medicine is also limited (Elrod and Hofmeister 2019; Grigg et al. 2019; Haake et al. 2023; Van Haaften et al. 2020). Since owners are ultimately responsible for the health and wellbeing of their pets, understanding owners' perceptions about medications and supplements will help to determine biases, misconceptions, or informational gaps. Because veterinarians often provide recommendations for pets' medications and supplements to clients, understanding the perceptions of final‐year veterinary students poised to enter the workforce may help to identify topics about which graduating students are well informed, uninformed, or misinformed. Understanding these perceptions may also inform educational curricula during veterinary training. Veterinary curricula in the United States are monitored by the American Association of Veterinary Medical Colleges (AAVMC), which has established the components of competency‐based veterinary medical education (CBVME), classified as Domains of Competence. Within Domain 1, Clinical Reasoning and Decision‐making, Competency 1.3 includes creating and adjusting a treatment plan based on available evidence and Competency 1.6 includes adapting knowledge to varied scenarios and contexts. Under Domain 2, Individual Animal Care and Management, Competency 2.2 requires the graduate to promote comprehensive wellness and preventative care (AAVMC 2024). The veterinary curriculum at the authors' institution in particular aims to have every graduate meet these outcomes, and as such, to educate our students about the benefits and limitations of management options, including administration of medications and supplements. Enhancing veterinarians' understanding of medications and supplements equips them with the knowledge to educate clients. The goal of this study was to determine Dog Aging Project newsletter recipients' perceptions of medications and supplements and compare those to the perceptions of final‐year veterinary students nearing graduation.

## 2 Materials and Methods

### 2.1 Newsletter Survey

To sample members of the general public interested in dogs about their perceptions, we surveyed recipients of the Dog Aging Project's newsletter. Briefly, the Dog Aging Project (DAP) is a large nationwide, longitudinal study of companion dogs (Creevy et al. 2022). The purpose of the DAP is to collect data and evaluate factors that influence aging in dogs, with goals of improving healthy longevity in dogs and, by translation, in humans (Creevy et al. 2022). Dogs are enrolled in the DAP by their owners, who complete various surveys and activities to provide data through individual password‐protected portals. The DAP utilizes an online helpdesk to support participants. Over time, the DAP team observed free‐text entries in data‐collection instruments addressing supplement use and tickets in the helpdesk system, which suggested lack of clarity about this term.

The DAP uses periodic newsletters to communicate with participants and non‐participant supporters, and occasionally includes short surveys in these newsletters. Newsletter survey responses are anonymous and are not linked to any DAP data about the respondent or their participating DAP dog (if any). To pursue the observations about lack of clarity regarding the term “supplements,” a newsletter survey was developed to invite respondents to indicate whether a list of attributes applied to a medication, a supplement or both. Attributes to include were generated from participant questions and free‐text comments, and from the clinical experiences of DAP veterinarians. An initial proposed list was circulated among veterinarians on the DAP team for refinement including eliminating redundancy, ensuring that phrasing was value‐neutral, and removing medical jargon that might imply that “medication” was the preferred response (e.g., the term “diagnosis” was replaced with “ailment”). Our survey was intentionally designed to ask separate questions about the use of medications and/or supplements for “ailments” and “conditions”, while avoiding the use of the term “disease,” or “diagnosis” which might be interpreted to mean a specific medical diagnosis requiring a specific medication. The refined survey was reviewed and revised by the DAP External Communications team to ensure clarity and ease of completion, and the final version was programmed into Google Forms. This final survey titled “Newsletter Survey – Medication or Supplement?” was received by 55,346 DAP newsletter recipients via Google Forms in July 2023. The survey remained open from July 26, 2023, to August 21, 2023, and consisted of 13 questions asking the respondent to categorize whether a statement was related to a medication, supplement, both, neither, or unsure (Table 1). An optional free‐response question was provided in which the respondent could type out anything else they wanted to share with the DAP team on this topic.

**TABLE 1:** Survey questions for dog‐interested newsletter recipients and final‐year veterinary students. Respondents chose a single response for each prompt.

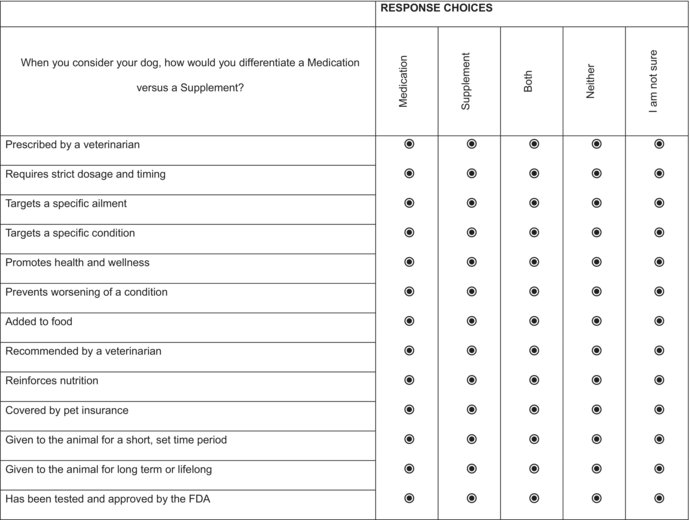

### 2.2 Survey for Final‐Year Veterinary Students

The newsletter survey was modified slightly before use with final‐year veterinary students at the Texas A&M University College of Veterinary Medicine and Biomedical Sciences (TAMU CVMBS). The original 13 questions from the newsletter survey were retained. Additionally, questions were added asking about the respondents' comfort level discussing medications and discussing supplements with clients, with responses captured using a 5‐point Likert‐type scale for which the anchors were “1 – Not comfortable at all” to “5 – Extremely comfortable.” Questions about the usefulness of sources from which they have learned about veterinary supplements were also added. The possible sources listed were “veterinary school coursework,” “extracurricular material offered at school,” “FDA website,” “word‐of‐mouth from mentors and/or veterinarians outside of vet school,” “manufacturer websites or outreach presentations,” “peer‐reviewed research,” and “consumer review websites.” For each source, the students could select one of the following responses: “not useful,” “a little bit useful,” “fairly useful,” “very useful,” or “I've never used this source of information.” The survey was sent to all final‐year students at TAMU CVMBS through an email listserv.

### 2.3 Statistical Analysis

We descriptively compared the results from the two groups with particular attention to the majority response selected by each group. Additionally, Fisher's exact tests were performed to determine whether the distribution of responses differed between the two groups, with the alpha level set at 0.05.

## 3 Results

A total of 1955 newsletter recipients (3.5% of 55,346) and 40 final‐year veterinary students (26% of the 152 students) responded to their respective surveys. Their responses are shown in Table 2.

**TABLE 2:** Percentage of responses from dog‐interested newsletter recipients (*n* = 1955) and final‐year veterinary students (*n* = 40) regarding perceptions of medications versus supplements.

	Medication %		Supplement %		Both %		Neither %		I am not sure %	
	Newsletter recipients	Final‐year veterinary students	Newsletter recipients	Final‐year veterinary students	Newsletter recipients	Final‐year veterinary students	Newsletter recipients	Final‐year veterinary students	Newsletter recipients	Final‐year veterinary students
Prescribed by a veterinarian	73	78	< 1	0	26	22	< 1	0	< 1	0
Requires strict dosage and timing	80	85	1	0	19	15	< 1	0	1	0
Targets a specific ailment	51	62	2	0	46	38	< 1	0	1	0
Targets a specific condition	40	58	5	2	54	40	< 1	0	1	0
Promotes health and wellness	3	0	51	38	44	62	1	0	1	0
Prevents worsening of a condition	25	22	13	5	60	72	1	0	1	0
Added to food	4	0	32	20	58	80	3	0	4	0
Recommended by a veterinarian	13	0	27	15	58	82	1	2	1	0
Reinforces nutrition	1	0	70	70	23	22	3	8	3	0
Covered by pet insurance	57	80	1	0	5	12	7	0	30	8
Given to the animal for a short, set time period	70	80	2	0	22	20	3	0	3	0
Given to the animal for long term or lifelong	12	2	28	20	55	72	3	5	2	0
Has been tested and approved by the FDA	77	85	3	0	13	15	2	0	5	0

*Note:* Data are rounded to whole numbers or to < 1% if the value rounded to 0.

There was a high concordance between groups of responses to three of the 13 questions in the survey. A majority, and a similar proportion, of newsletter recipients (73%) and final‐year veterinary students (78%) indicated that only medications and not supplements are prescribed by a veterinarian. The majority, and a similar proportion, of both groups also indicated that only medications require strict dosing and timing (80% of newsletter recipients and 85% of final‐year veterinary students). Also, the majority (70% in each group) indicated that only supplements reinforce nutrition.

For eight questions, the majority of each group chose the same response, but the frequency of that majority choice differed by greater than 10% (Figure 1). This figure was constructed to illustrate the topics for which there is a difference in the majority response between newsletter recipient and student responses, as these potentially represent areas about which veterinarians may need to educate clients. A majority of both groups perceived that only medications target a specific ailment (51% newsletter recipients; 62% final‐year veterinary students), that both medications and supplements prevent worsening of a condition (60% newsletter recipients; 72% final‐year veterinary students), and that both medications and supplements are added to food (58% newsletter recipients; 80% final‐year veterinary students). A majority of both groups indicated that both medications and supplements are recommended by veterinarians (58% newsletter recipients; 82% final‐year veterinary students), that only medications are covered by pet insurance (57% newsletter recipients; 80% final‐year veterinary students), that only medications are used short‐term (70% newsletter recipients; 80% final‐year veterinary students), and that both medications and supplements are given long term (55% newsletter recipients; 72% final‐year veterinary students). Lastly, a majority of both groups chose only medications as being FDA tested and approved (77% newsletter recipients; 85% final‐year veterinary students). Notably, the remaining 15% of final‐year veterinary students indicated that both medications and supplements are FDA tested and approved. Among these eight items, the overall distribution of responses was statistically significantly different between groups for three attributes: added to food (*p* < 0.001); recommended by a veterinarian (*p* = 0.005); and covered by pet insurance (*p* < 0.001).

**FIGURE 1:**
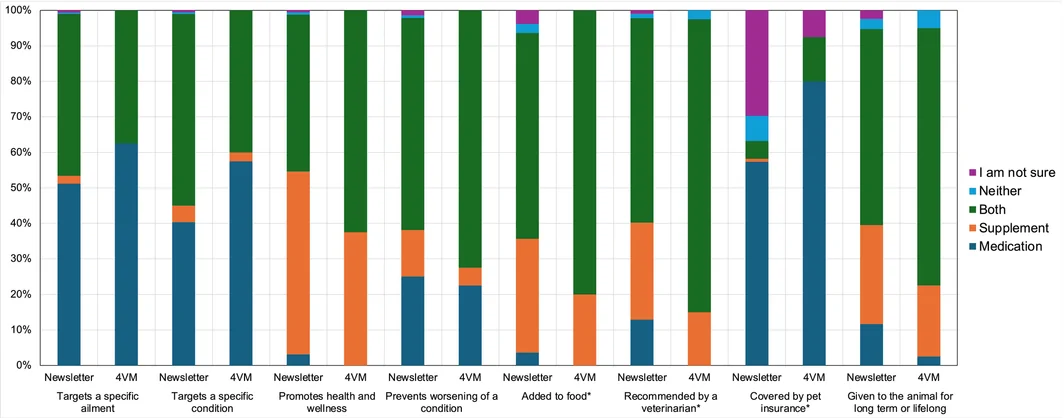
Perceptions of medications and supplements for which the majority response differed between dog‐interested newsletter recipients of the general public and final‐year veterinary students at Texas A&M University. *Overall distribution of responses is different between groups at p < 0.05 using Fisher’s Exact Test.4VM, Final‐year veterinary medical students; Newsletter, Dog‐interested newsletter recipients.

For two questions, the majority of each group did not choose the same response. Majority responses were different regarding whether medications and/or supplements target a specific condition, with 54% of newsletter recipients choosing both medications and supplements and 58% of final‐year veterinary students choosing only medications. Majority groups were also different regarding which products promote health and wellness, with 51% of newsletter recipients choosing only supplements and 62% of final‐year veterinary students choosing both medications and supplements.

In general, final‐year veterinary students reported higher levels of comfort answering client questions about medications than supplements (Figure 2). The information sources that students most frequently reported as very useful were peer‐reviewed research and word‐of‐mouth from mentors or veterinarians outside of vet school (Figure 3).

**FIGURE 2:**
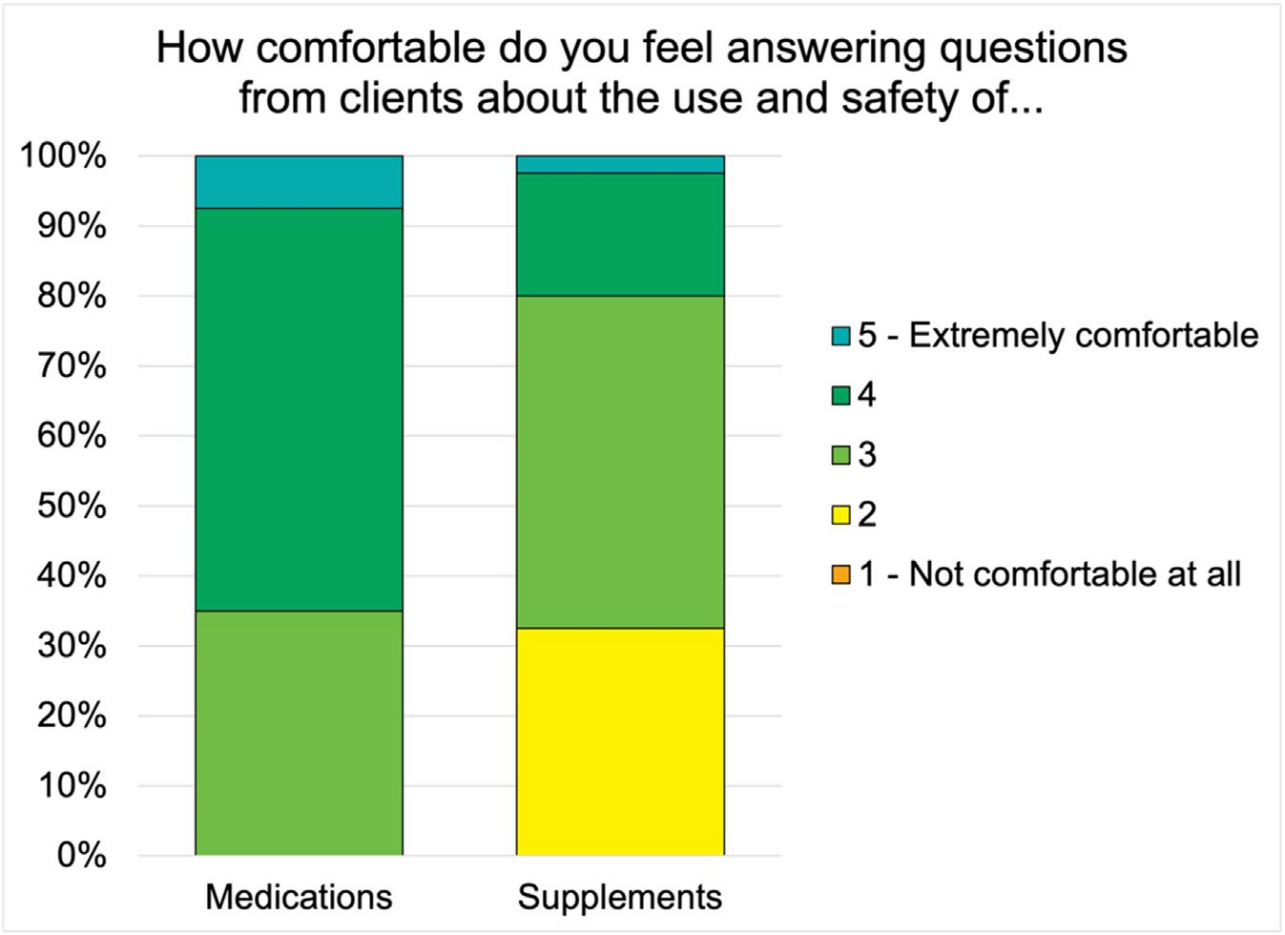
Final‐year veterinary students' (*n* = 40) level of comfort answering questions about medications and supplements.

**FIGURE 3:**
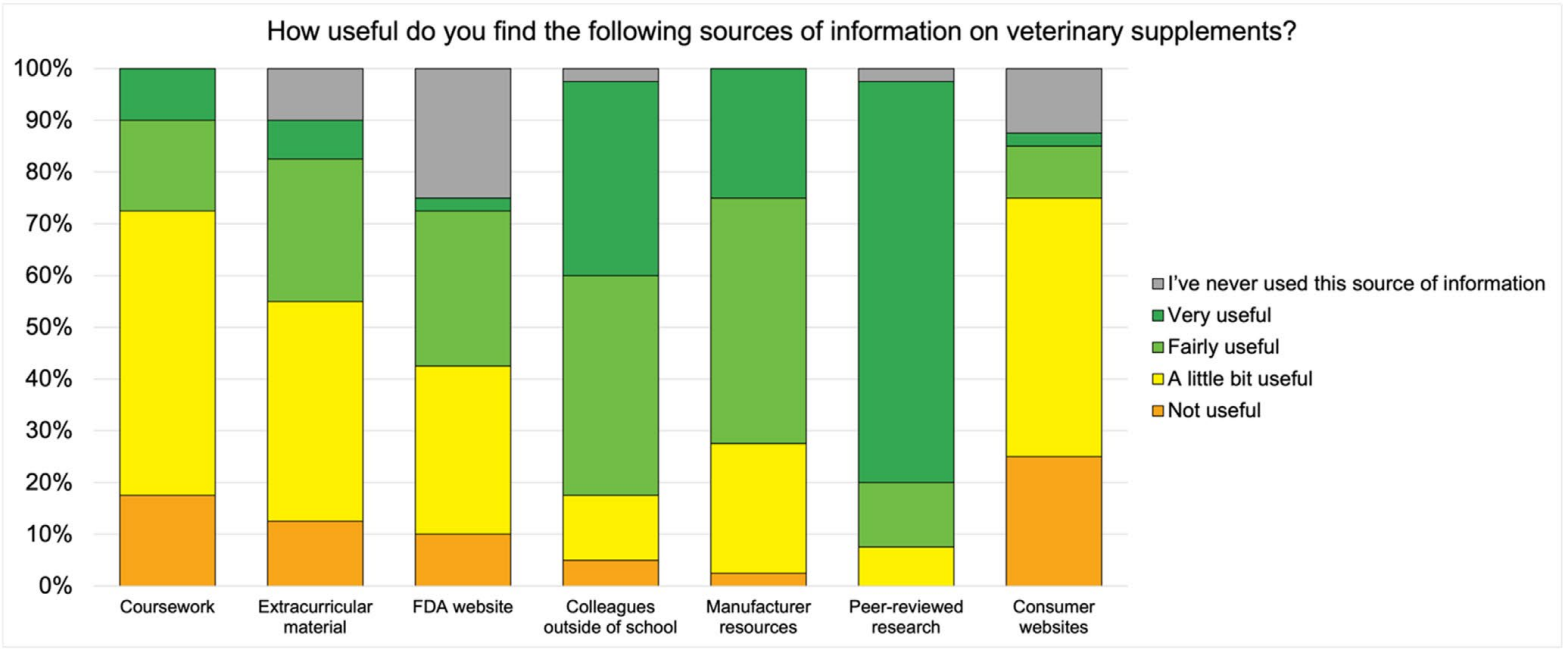
Final‐year veterinary students' (*n* = 40) opinions on the usefulness of resources about supplements.

## 4 Discussion

This study provides information about the perceptions of medications versus supplements, and how those might differ among various audiences. We considered these “perceptions” since, given that there is not a consistent legal definition that can be applied to all veterinary supplements, for some items in our survey there was no single correct answer. In our study, a majority of newsletter recipients, representing the dog‐interested general public, and final‐year veterinary students recognized that only medications, and not supplements, are tested and approved by the FDA. However, among newsletter recipients, a total of 16% indicated that supplements are subject to FDA approval (either in conjunction with medications, or alone) and another 5% indicated that they were not sure. The finding parallels findings from human medical research that consumers (Dodge et al. 2011) and medical residents (Ashar et al. 2007) inaccurately believed that the contents of supplements are analyzed by the FDA and that supplements are tested for safety and efficacy, and are regulated by the FDA. Importantly, 15% of final‐year veterinary students also inaccurately reported that *both* medications and supplements are FDA regulated. This may indicate an educational gap resulting from the fact that supplements are not routinely overtly addressed in veterinary school curricula (Schoen 2000) and may be a target for improvement in the curriculum at the authors' institution of the TAMU CVMBS.

Considering the role of the veterinarian in influencing the use of these products, a majority of newsletter recipients and final‐year veterinary students indicated only medications are *prescribed* by a veterinarian, while a majority of both groups indicated that both medications and supplements are *recommended* by a veterinarian. The term “prescribing” may be associated with notions of a legal framework, whereas “recommending” may not be. In human medicine, a prescription is defined as a legal document that authorizes the patient to receive a substance that has the potential to be harmful if used without supervision of an authorized medical provider (Miller and Nicolas 2024). A number of situations may lead to confusion with this term. For example, sometimes a vitamin or mineral available as a supplement may be prescribed to a patient for a diagnosed vitamin or mineral deficiency. Veterinary use of human over‐the‐counter (OTC) pharmaceuticals is extralabel, so it could be considered to require a prescription (FDA—CFR 2024). However, many veterinarians do not write a prescription for extralabel OTC medications and instead provide dosing recommendations and advise the client to procure the product from an outside source.

Naturalness bias, or the belief that natural products (which often include supplements) are inherently better, safer, and healthier than their synthetic counterparts (Ji et al. 2023), can be observed in both human and veterinary medicine. Previous research indicates that supplements are perceived as safe because they are seen as food and are typically sourced from natural substances (Chiba and Tanemura 2022). Similarly, it has been reported that supplements are perceived to have fewer adverse effects than medications (Goodman and Trepanier 2005).

A majority of respondents perceived that only medications require strict dosing and timing, implying that both groups believed supplements do *not* require strict dosing and timing. However, adverse effects, overdoses, and drug interactions can occur with supplements, and thus the dosing and timing of certain supplements are necessary to avoid undesired outcomes (Goodman and Trepanier 2005; Nobles and Khan 2015; The Official Top 10 Pet Toxins of 2023 | ASPCA 2023). Notably, in a 2019 report, veterinarians listed safety and efficacy as the most important factors in deciding the use of supplements (referred to as nutraceuticals in that study) in clients' and personal pets (Elrod and Hofmeister 2019). The importance of proper dosing may represent another area that requires further emphasis in our curriculum so that veterinarians can properly guide clients' administration of supplements.

As mentioned, the survey was intentionally designed not to use the terms “disease” or “diagnosis” to avoid the implication of a definitive diagnosis or prescribed therapy, both of which might be linked to the concept of “medication.” Survey results suggest that newsletter recipients and final‐year veterinary students seemed to have different interpretations of the terms ailment and condition. The majority of both groups perceived that only medications target a specific “ailment.” In contrast, the majority of final‐year veterinary students perceived that only medications target specific conditions, while the majority of newsletter recipients perceived that both medications and supplements target specific conditions. While supplements cannot be labeled to indicate they treat, cure, mitigate, or prevent disease, they can be labeled with health claims, nutrient content claims, or structure/function claims (Label Claims for Food and Dietary Supplements | FDA 2024). Structure/function claims may suggest that a supplement improves health of an organ system (Label Claims for Food and Dietary Supplements | FDA 2024), thereby “targeting a specific condition.” Veterinarians may need to assist clients in understanding the label claims of supplements.

A similar majority of both groups indicated that medications are given for a short period. A majority of both groups also indicated that both medications and supplements are given long term or lifelong, although there was a 17% higher frequency of this majority response among final‐year veterinary students. While respondents may associate medications with the treatment of acute illnesses, the duration of usage of both medications and supplements actually depends on the indication. A previous DAP study evaluating owner‐reported medical conditions showed that the most frequently reported conditions in this population included dog bite, giardiasis, seasonal allergies, and ear infection, which are often acute conditions (Forsyth et al. 2023). Many participating dog owners had dogs with no apparent chronic conditions (Forsyth et al. 2023). Therefore, dog owners may first think about the more common acute conditions when asked about medication for their dogs. Veterinary students are likely more familiar with chronic conditions that require long‐term treatment, which may explain the difference in frequency of the majority response about long‐term usage.

Wellness is a subjective term without a universal definition, especially as it relates to pets. A majority of newsletter recipients associated only supplements with promoting health and wellness; in contrast, most final‐year veterinary students associated both medications and supplements with promoting health and wellness. This suggests that our curriculum educates students about the beneficial effects of medications on patient health, and that this is a topic that veterinarians may need to explain to clients. Veterinary students are taught that preventative medications, such as those against heartworms, fleas, and ticks, are classified as medications. Because dog owners may not think about preventives when the term medication is applied, students are instructed throughout the curriculum to inquire about preventives separately from asking about current medications when taking a medical history. By contrast, clients may interpret the need for a medication as indicating that a dog is unwell, rather than viewing the medication as a means of restoring or maintaining the dog's health.

To evaluate reasons that veterinary students hold certain perceptions about supplements or medications, we asked them about their sources of information on each. Many curricula do not incorporate extensive education about supplements, so veterinary students may be most familiar with recommendations for medications (Schoen 2000). In our survey, the majority of final‐year veterinary students cited primary literature and word‐of‐mouth recommendations from mentors and veterinarians outside of the curriculum as being the most useful resources for information about supplements, which may support the need for increased attention to supplements in our curriculum to align with CBVE competencies for new graduates. Previously, veterinarians who were surveyed reported scientific articles and manufacturer‐provided information as their major sources for information about supplements (Elrod and Hofmeister 2019). Other reports have found that the dog‐owning public and veterinarians use social media groups and blogs about pet health topics, pet nutrition topics, and nutrition as important sources of information about these products (Bianco et al. 2020).

There are several limitations with this study. The newsletter recipients surveyed may not be representative of the general dog‐owning population because the survey was conducted through DAP. Additionally, because the newsletter survey was submitted voluntarily and anonymously by newsletter recipients, we did not collect demographic information about the respondents. Because newsletter recipients include DAP research participants and other interested people, we cannot extrapolate the demographic information from the DAP participants to the respondents of this survey. While DAP participants are a large, nationwide population, it is possible that people interested in the DAP are more interested in dog health than average Americans, which could mean that they are more educated about products such as medications and supplements. It is also possible that respondents interpreted questions variably, and/or differently from how the authors had intended. For instance, a substance such as a vitamin may be used to supplement a diet or may be used to manage a medical deficiency, making the categorization as a supplement or medication unclear, particularly as pertains to the question about products that reinforce nutrition. Additionally, some of the newsletter recipients may have been veterinarians participating in the DAP project, which could impact their knowledge of supplements and medications compared to non‐veterinarian owners. Finally, newsletter recipients were not asked about why they held the beliefs indicated by their responses, and so it is not possible to comment on the source(s) of their information. Another limitation was the small final‐year veterinary student sample size. Final‐year veterinary students who were within 1 month of graduation were selected to represent the day‐one veterinarian, which is the emphasis of the Texas A&M University veterinary curriculum. However, the students who responded may not be representative of the entire final‐year veterinary student population. For instance, they may be more interested and informed about the management of small animal health and disease than the average final‐year student. Additionally, the student population was derived from a single school (Texas A&M University), and it is possible that students from other veterinary schools would have responded differently based on differing curricula. For these reasons the statistical comparisons between groups should be interpreted cautiously and used to suggest areas for further research with targeted groups, to determine whether and why such differences persist.

## 5 Conclusions

Perceptions about what is meant by the terms “medication” and “supplement” vary between members of the dog‐interested public and final year veterinary students. Opportunities exist to improve graduating veterinary students' knowledge and understanding of supplements and medications in order to enhance future veterinarians' ability to convey accurate information to clients when discussing treatment options for veterinary patients.

## Author Contributions

S.T.G., J.C.H., K.I., V.R.F., and K.E.C. contributed meaningfully to the study and manuscript. The Dog Aging Project consortium is an integral part of making the project possible.

## Funding

This research is based on publicly available data collected by the Dog Aging Project, under U19 grant AG057377 (PI: Daniel Promislow) from the National Institute on Aging, a part of the National Institutes of Health, and by additional grants and private donations, including generous support from the Glenn Foundation for Medical Research, the Tiny Foundation Fund at Myriad Canada, and the WoodNext Foundation. These data are housed on the Terra platform at the Broad Institute of MIT and Harvard. Authors K.E.C., V.R.F., and K.I. received funding from U19 AG057377.

## Ethics Statement

No ethical approval was required as animals were not used in this study. The University of Washington IRB deemed that recruitment of dog owners for the Dog Aging Project and the administration and content of the DAP Health and Life Experience Survey (HLES) are human subjects research that qualifies for Category 2 Exempt status (IRB ID number 5988, effective 10/30/2018). Additionally, the use of newsletters to communicate with DAP participants and gather anonymous information from them that facilitates the DAP's research conforms to this Exempt status. The student survey was determined to be exempt by the Texas A&M University IRB committee (STUDY2024‐0103, exemption in accordance with 45 CFR 46.104).

## Conflicts of Interest

The authors declare no conflicts of interest.

## Data Availability

The data that support the findings of this manuscript will be made available upon request from the corresponding author.
